# Soy consumption and risk of COPD and respiratory symptoms: a case-control study in Japan

**DOI:** 10.1186/1465-9921-10-56

**Published:** 2009-06-26

**Authors:** Fumi Hirayama, Andy H Lee, Colin W Binns, Yun Zhao, Tetsuo Hiramatsu, Yoshimasa Tanikawa, Koichi Nishimura, Hiroyuki Taniguchi

**Affiliations:** 1 School of Public Health, Curtin University of Technology, Perth, WA, Australia; 2 Department of Respiratory Medicine and Allergy, Komaki City Hospital, Aichi, Japan; 3 Department of Respiratory Medicine and Clinical Immunology, Toyota Kosei Hospital, Aichi Prefectural Welfare Federation of Agricultural Cooperatives, Aichi, Japan; 4 Department of Respiratory Medicine, Murakami Memorial Hospital, Asahi University, Gifu, Japan; 5 Department of Respiratory and Allergic Medicine, Tosei General Hospital, Aichi, Japan

## Abstract

**Background:**

To investigate the relationship between soy consumption, COPD risk and the prevalence of respiratory symptoms, a case-control study was conducted in Japan.

**Methods:**

A total of 278 eligible patients (244 men and 34 women), aged 50–75 years with COPD diagnosed within the past four years, were referred by respiratory physicians, while 340 controls (272 men and 68 women) were recruited from the community. All participants underwent spirometric measurements of respiratory function. Information on demographics, lifestyle characteristics and habitual food consumption was obtained using a structured questionnaire.

**Results:**

Total soy consumption was positively correlated with observed lung function measures. The mean soy intake was significantly higher among controls (59.98, SD 50.23 g/day) than cases (44.84, SD 28.5 g/day). A significant reduction in COPD risk was evident for highest versus lowest quartile of daily intake of total soybean products, with adjusted odds ratio (OR) 0.392, 95% CI 0.194–0.793, *p *for trend 0.001. Similar decreases in COPD risk were associated with frequent and higher intake of soy foods such as tofu and bean sprouts, whereas respiratory symptoms were inversely associated with high consumption of soy foods, especially for breathlessness (OR 0.989, 95% CI 0.982–0.996).

**Conclusion:**

Increasing soy consumption was associated with a decreased risk of COPD and breathlessness.

## Background

Chronic obstructive pulmonary disease (COPD) is a leading cause of morbidity and mortality worldwide [1], with cigarette smoking being established as the principle risk factor [2-4]. However, while 95% of COPD patients are, or have been, cigarette smokers, only 20% of smokers develop COPD [5]. Therefore, other factors such as dietary and environmental exposures may protect against, or contribute to, disease development. Because of the high burden and societal cost of COPD, studies on potential new methods of prevention through an appropriate diet are important. An extensive literature review suggested that fruits, vegetables, fish, and meat may be associated with the risk of COPD and respiratory symptoms [6].

High fruit intake is inversely related to the COPD risk [7-9] and respiratory symptoms [10-12]. A cohort study of 49,140 Singaporean men observed that non-citrus fruits (apples, pears and grapes) were independently associated with reduced cough with phlegm [12]. Similarly, a cross-sectional study among 13,651 adults aged 20–59 years in The Netherlands reported that solid fruits (apples and pears) were inversely associated with the prevalence of COPD symptoms such as chronic cough and breathlessness [10]. The same authors also noted the favourable effect of fruits (> 180 g/day) in lowering the prevalence of these respiratory symptoms [11]. Increased vegetable consumption is likely to reduce the risk of COPD [7,8,13]. However, two large studies conducted in The Netherlands and Singapore observed no relationship between vegetable intake and the prevalence of respiratory symptoms [11,12].

One cross-sectional study from Norway involving 4,300 subjects found fish intake beneficial against developing a night cough but not other symptoms [14]. On the other hand, epidemiological evidence from several prospective studies indicated that red meat or cured meat consumption had detrimental effects on the risk of COPD [15-18]. In particular, preserved red meat was positively associated with respiratory symptoms [19] and could lead to a decline in lung function [17].

Little is known in relation to soy foods, apart from one cohort study in Singapore which suggested that soy foods may reduce the development of chronic productive cough [12], and conclusions cannot be drawn because of the limited evidence [6].

Soybean products, a primary source of isoflavones, are consumed worldwide. In Japan, a wide variety of soy foods are available including tofu (soybean curd), natto (fermented soybeans), miso (fermented soybean paste) soup, bean sprouts and soy milk. The aim of this study was to investigate the relationship between soy consumption and the risk of COPD and respiratory symptoms.

## Methods

### Study design and subjects

A case-control study was conducted in central Japan in 2006. Three hundred COPD patients referred by respiratory physicians were recruited from the outpatient departments of six hospitals in Aichi, Gifu and Kyoto. According to the protocol of the Global Initiative for Chronic Obstructive Lung Disease (GOLD) [20], diagnosis of COPD was confirmed by spirometry with FEV_1_/FVC < 0.7, where FEV_1 _= forced expiratory volume in one second and FVC = forced vital capacity. Predicted FEV_1 _was calculated using the Japanese Respiratory Society's Guidelines [21].

Inclusion criteria for cases were: (i) age between 50 and 75 years; (ii) had COPD as the primary functionally limiting illness which was diagnosed within the past four years. Subjects were excluded if they had a recent stroke, dementia or other health conditions that prohibited them from being interviewed. Twenty-two eligible patients were subsequently excluded due to missing or incomplete demographic and lifestyle details. The remaining 278 patients (244 men and 34 women) were available for analysis. No statistically significant differences were found between the included and excluded prevalent cases in terms of mean age, BMI and gender distribution (p > 0.05). Permission to recruit patients and access to medical records were granted by the participating hospitals in Japan.

During the same period, 400 community-dwelling adults were recruited from the same catchment areas as the prevalent cases. These controls were approached and interviewed at shopping malls, community centres or when they attended health checks at hospitals. They were selected to be frequency matched to the prevalent cases by age (± 5 years). The same exclusion criteria as cases were applied, resulting in 340 eligible controls (272 men and 68 women). All participants underwent spirometric measurements of respiratory function to avoid misclassification of case-control status. Approval of the study protocol was obtained from the Human Research Ethics Committee of Curtin University (approval number HR 90/2005) and the six hospitals in Japan.

### Interview and questionnaire

A structured questionnaire was administered face-to-face by the first author to collect information from each participant. Demographic and lifestyle characteristics solicited included age, gender, weight (kg), height (m), education level (high school or below; college or university), cigarette smoking (never smoker; ex-smoker; current smoker) and alcohol drinking status (non-drinker; drinker). For the prevalent cases, each interview was conducted in the presence of their next-of-kin to minimize recall error, and appointment was made via their respiratory physician. The purpose of the study was explained to each participant before obtaining their formal written consent. Confidentiality of the information provided, and the right to withdraw without prejudice, were ensured and maintained throughout the study. All interviews, averaging 30 minutes in duration, took place in the hospital outpatient departments for COPD patients and their place of recruitment for controls.

Information on habitual food consumption was obtained using a 138-item interviewer-administered food frequency questionnaire taken from the Japan Public Health Center-based prospective study on cancer and cardiovascular disease [22]. Its validity and reproducibility had been established for the Japanese population [23]. The reference recall period for dietary variables was set at 5 years before interview. Therefore, the recall period was shorter among cases (before their diagnoses of COPD) and different from case to case depending on their date of diagnosis. Soy food consumption encompassed tofu (boiled or cold, in miso soup, freeze-dried, deep-fried), natto, bean sprouts, and soy milk which are soybean products. The frequency of intake of tofu, natto and bean sprouts was classified by nine categories: 'almost never', 'once to three times per month', 'once to twice per week', 'three to four times per week', 'five to six times per week', 'once per day', 'twice to three times per day', 'four to six times per day', and '7 or more times per day'. Those participants who answered "monthly" to "daily" were asked their usual amount consumed per meal in terms of standard portion size (tofu, 75 g; tofu in soup, 25 g; dried tofu, 60 g; deep-fried tofu, 2 g; natto, 75 g; bean sprouts, 25 g). The frequency of soy milk drinking was categorized into 'almost never', '1 to 2 times per week', '3 to 4 times per week', '5 to 6 times per week', 'one cup per day', '2 to 3 cups per day', '7 to 9 cups per day', and '10 or more cups per day.'

A further question on 'life-long physical activity involvement' was appended to the questionnaire, defined as "doing active sports or vigorous exercise long enough to get sweaty, at least twice a week", over the entire life course [24]. Response options were dichotomous: 'has never been involved to not any more involved in such activity' and 'has always been involved in such activity'.

Two screening instruments, Medical Research Council's "dyspnoea" scale [25] and the Australian Lung Foundation's "Feeling Short of Breath" scale [26], were used to assess respiratory symptoms of each individual. The latter scale consisted of five simple questions: (i) Do you cough several times most days? (ii) Do you bring up phlegm or mucous most days? (iii) Do you get out of breath more easily than others your age? (iv) Are you over 40 years old? (v) Are you smoker or ex-smoker?

### Statistical analysis

Descriptive statistics were first applied to summarise participant characteristics and lung function measures. The daily intake of soy products (g) was derived from the frequency and quantity recorded, accounting for the edible portion of each food. After comparing the dietary consumption pattern between case and control groups, multivariate (unconditional) logistic regression analyses were performed to assess the effects of soy foods on the COPD risk and the prevalence of respiratory symptoms. Soy consumption variables were further categorised based on the corresponding empirical distribution of controls, with the lowest intake or none being the reference category. Besides soy foods consumption, independent variables included in the regression models were age, gender, body mass index (BMI = weight/height^2^) of five years ago, education level, life-long physical activity involvement, smoking status, smoking pack-years, alcohol drinking status and daily dietary intake of fruits, vegetables, fish, red meat and chicken. These variables were considered potential confounders from the literature. Adequacy of each fitted model was assessed by the Hosmer-Lemeshow statistic as well as area under the ROC curve. All statistical analyses were undertaken using the SPSS for Windows package version 13.

## Results

Table 1 presents the demographic and lifestyle profile of the participants by gender and case-control status. The average age of subjects was about 66 years. For the prevalent case group, 220 patients (80%) had their COPD diagnosed within the past two years. The mean BMI (five years ago) of COPD patients was lower than that of controls. The majority of participants had high school or below education and were seldom involved in physical activity over the life course. A substantial proportion of COPD patients (21.8% for male and 27.3% for female) continued to smoke after their diagnosis of COPD.

**Table 1:** Characteristics of COPD patients and control subjects

**Variable**	**Cases**		**Controls**	
	Male (n = 244)	Female (n = 34)	Male (n = 272)	Female (n = 68)
Mean age (years)	66.51 (SD 6.82)	66.10 (SD 6.13)	65.15 (SD 5.41)	66.12 (SD 5.76)
Mean BMI five years ago (kg/m^2^)	22.09 (SD 2.94)	20.67 (SD 3.89)	23.61 (SD 2.85)	23.30 (SD 3.25)
Education: High school or below	195 (80.2%)	26 (78.8%)	166 (61.9%)	47 (70.1%)
Life-long physical activity: Never to not any more involved	185 (75.8%)	29 (85.3%)	182 (68.2%)	56 (82.4%)
Alcohol drinkers	150 (61.5%)	8 (23.5%)	202 (74.5%)	21 (30.9%)
Cigarette smoking status				
Ex-smokers	188 (77.4%)	20 (60.6%)	133 (49.1%)	3 (4.4%)
Current smokers	53 (21.8%)	9 (27.3%)	63 (23.2%)	2 (2.9%)
Mean smoking (pack-years)	65.03 (SD 24.91)	43.25 (SD 31.67)	30.90 (SD 28.81)	2.04 (SD 9.68)
FEV_1_	1.64 (0.69)	1.15 (0.47)	2.56 (0.51)	1.76 (0.35)
FVC	3.08 (0.83)	2.07 (0.52)	3.31 (0.60)	2.17 (0.41)
FEV_1_/FVC (%)	52.70 (14.71)	55.30 (14.47)	78.06 (5.96)	81.74 (5.66)
FEV_1_% predicted	56.68 (22.32)	56.52 (19.40)	86.54 (14.04)	93.85 (13.76)

The overall prevalence of respiratory symptoms was: cough (n = 105, 17%); sputum (n = 193, 31%); breathlessness (n = 217, 35%), with significantly higher prevalence in the COPD group (*p *< 0.001), especially for breathlessness (66% in cases versus 12% in controls). The binary breathlessness outcome was validated against the Medical Research Council's dyspnoea score. The observed spearman rank correlation of 0.7 confirmed a good agreement between the two scales. Distribution of the dyspnoea scale was: none, 46.9%; Grade 1, 23%; Grade 2, 18.5%; Grade 3, 7.8%; Grade 4, 3.1%; Grade 5, 0.7%.

Table 2 shows the dietary pattern of participants five years ago. The COPD patients had lower daily intake of fruits, vegetables, chicken and fish than subjects without the disease, except red meat. In particular, the mean soy intake was significantly lower among the prevalent cases (44.84, SD 28.50 g/day) than controls (59.98, SD 50.23 g/day), *p *< 0.001. Table 3 further contrasts the frequency of soy consumption between the two groups, which clearly indicates the less frequent eating of soybean foods by COPD patients. Only a few participants drank soy milk, albeit the significant difference in frequency distribution between COPD patients and controls.

**Table 2:** Dietary intake by participants

**Dietary intake** g/day, mean (SD)	**Cases** (n = 278)	**Controls** (n = 340)	*p *value (*t *test)
Fruits	218.36 (175.68)	270.34 (239.88)	0.002
Vegetables	185.49 (101.85)	224.39 (140.29)	< 0.001
Red meat	40.63 (35.31)	34.55 (27.01)	0.019
Chicken	10.33 (13.73)	12.43 (13.55)	0.058
Fish	19.54 (21.95)	22.04 (25.72)	0.200
Total soy foods*	44.84 (28.50)	59.98 (50.23)	< 0.001
Tofu	17.66 (16.45)	25.02 (26.24)	< 0.001
Natto	11.43 (15.58)	18.03 (33.51)	0.001
Bean sprouts	3.88 (5.02)	4.59 (4.61)	0.066
Soy milk (cups/week)	0.19 (1.01)	0.46 (1.74)	0.016

* Defined as the intake of solid soy foods comprising tofu (boiled or cold, in miso soup, freeze-dried, deep-fried), natto (fermented soybean), and bean sprouts.

**Table 3:** Frequency of soy foods intake by participants

**Soy food**	**Cases** (n = 278)	**Controls** (n = 340)	*p *value (chi-square test)
Tofu*			0.016
Never	31 (11.2%)	27 (8.0%)	
1 to 3 times/month	93 (33.6%)	96 (28.6%)	
1 to 6 times/week	148 (53.4%)	192 (57.1%)	
At least once/day	5 (1.8%)	21 (6.3%)	
Bean sprouts*			< 0.001
Never	73 (26.3%)	58 (17.3%)	
1 to 3 times/month	88 (31.7%)	81 (24.1%)	
1 to 6 times/week	112 (40.3%)	192 (57.1%)	
At least once/day	5 (1.8%)	5 (1.5%)	
Natto*			0.001
Never	107 (38.5%)	84 (24.9%)	
1 to 3 times/month	39 (14.0%)	41 (12.2%)	
1 to 6 times/week	101 (36.3%)	155 (46.0%)	
At least once/day	31 (11.2%)	57 (16.9%)	
Soy milk*			0.008
Never	265 (95.3%)	300 (89.6%)	
At least once/week	13 (4.7%)	35 (10.4%)	

* missing data present

The relationship with lung function was next examined. [see Figure S1, Additional file 1] presents the scatter plot of FEV_1 _% predicted against total soy intake. All observed lung function measures (FVC, FEV_1_, FEV_1_/FVC and FEV_1 _% predicted) were positively correlated with total soy consumption. Linear regression results in [Table S1, Additional file 1] further confirmed the significant positive association between lung function and soy intake even after adjusting for the effects of confounding factors.

Table 4 summarizes the results from logistic regression analyses with respect to soy foods consumption. A significant reduction in COPD risk was evident for the highest versus lowest quartile of daily total intake of soybean products, with adjusted odds ratio (OR) 0.392, 95% CI 0.194–0.793. The corresponding test for trend was highly significant (*p *= 0.001). Similar decreases in COPD risk were associated with higher intakes of tofu and bean sprouts, but to a lesser extent for natto. As expected, the effect of soy milk did not attain statistical significance because of the small number of drinkers. To assess the sensitivity of the analysis, logistic regressions were rerun for the subset of 157 patients with COPD diagnosed within one year. The additional results [Table S2, see Additional file 1], were generally consistent with those of all 278 prevalent cases. Moreover, very few interaction terms were found to be significant, so that effect modifications in these models should be minimal. Finally, goodness-of-fit of the models in Table 4 was assessed by the Hosmer-Lemeshow statistic, with corresponding *p *values 0.064, 0.168, 0.299, 0.813 and 0.052. The area under the ROC curve ranged between 0.881 and 0.887, further indicating adequacy of these fitted models.

**Table 4:** Soy consumption and COPD risk

**Variable**	**Cases** n (%)	**Controls** n (%)	OR*	**95% CI**	*p *value
**Total soy foods (g/day)**					*p *overall = 0.043 *p *trend = 0.001
≤ 30.43	95 (34.2)	85 (25.1)	1		
30.44 – 50.42	88 (31.7)	85 (25.1)	0.764	(0.421, 1.386)	
50.43 – 75.82	57 (20.5)	85 (25.1)	0.534	(0.284, 1.002)	
≥ 75.83	38 (13.7)	84 (24.8)	0.392	(0.194, 0.793)	
**Tofu (g/day)**					*p *overall = 0.019 *p *trend = 0.001
≤ 7.53	90 (32.4)	89 (26.2)	1		
7.54 – 18.05	98 (35.3)	87 (25.6)	0.997	(0.559, 1.777)	
18.06 – 28.68	50 (18.0)	79 (23.2)	0.735	(0.384, 1.406)	
≥ 28.69	40 (14.4)	85 (25.0)	0.378	(0.190, 0.753)	
**Bean sprouts (g/day)**					*p *overall = 0.003 *p *trend = 0.112
≤ 1.67	84 (30.2)	70 (20.6)	1		
1.68 – 5.34	84 (30.2)	90 (26.5)	0.454	(0.240, 0.856)	
5.35 – 5.36	64 (23.0)	107 (31.5)	0.302	(0.160, 0.570)	
≥ 5.37	46 (16.5)	73 (21.5)	0.374	(0.180, 0.777)	
**Natto (g/day)**					*p *overall = 0.295 *p *trend = 0.178
Never	108 (38.8)	88 (25.9)	1		
≤ 10.70	103 (37.1)	131 (38.5)	0.625	(0.362, 1.079)	
10.71 – 39.30	38 (13.7)	68 (20.0)	0.637	(0.322, 1.260)	
≥ 39.31	29 (10.4)	53 (15.6)	0.578	(0.280, 1.193)	
**Soy milk**					*p *overall = 0.143 *p *trend = 0.568
Never	265 (95.3)	300 (88.2)	1		
weekly drinkers	8 (2.9)	20 (6.0)	0.340	(0.116, 0.993)	
daily drinkers	5 (1.8)	15 (4.5)	0.927	(0.232, 3.707)	

* Adjusted odds ratios from logistic regression models including age, gender, BMI (5 years ago), education level (high school or below; college or university), alcohol drinking (non drinker; drinker), cigarette smoking (never smoker; ex-smoker; current smoker), smoking pack-years, life-long physical activity involvement (never to not any more involved; always been involved), daily intake of red meat, chicken, fish, vegetables and fruits.

Soy consumption was also associated with the presence of respiratory symptoms according to chi-square tests, and negatively correlated with the dyspnoea scale (r = -0.152). As shown in Table 5, participants presented with cough and breathlessness ate significantly less soy foods per day than others without the symptoms. Logistic regression results further confirmed an inverse relationship between total daily soy intake and the prevalence of breathlessness, with a small but significant 1% reduction in risk associated with each additional gram of intake. Effects concerning individual soy foods were similar and thus their logistic regression results were omitted for brevity.

**Table 5:** Soy consumption and respiratory symptoms

**Respiratory symptom**	**Total soy foods** mean g/day (SD)	OR *	**95% CI**	*p *value
Cough				0.051
No	55.26 (45.28)	1		
Yes	43.95 (24.61)	0.991	(0.983, 1.000)	
Sputum				0.417
No	53.34 (46.19)	1		
Yes	53.20 (33.88)	1.002	(0.997, 1.007)	
Breathlessness				0.002
No	58.94 (48.38)	1		
Yes	43.20 (26.97)	0.989	(0.982, 0.996)	

* Adjusted odds ratios from logistic regression models including age, gender, BMI (5 years ago), education level (high school or below; college or university), alcohol drinking (non drinker; drinker), cigarette smoking (never smoker; ex-smoker; current smoker), smoking pack-years, life-long physical activity involvement (never to not any more involved; always been involved), daily intake of red meat, chicken, fish, vegetables and fruits.

## Discussion

In this case-control study, the effects of soy bean products on lung function, COPD risk and the prevalence of respiratory symptoms were documented. All participants were carefully screened for respiratory symptoms and spirometric measurements of lung function were taken to ensure correct classification. Another strength of the study was the inclusion of mainly patients (80%) with COPD diagnosed within the past two years and all 278 patients were interviewed within four years of confirmed COPD diagnosis, which provided better accuracy in capturing their dietary exposure information. Moreover, the total intake of soy foods by our community-dwelling controls (mean 59.98 g/day, SD 50.23) was comparable with the average consumption level of 60.4 g/day by the general Japanese population in 2006 [27].

Soy consumption was found to be positively correlated with lung function and inversely associated with the risk of COPD. The epidemiological evidence also indicated an inverse association between total soy intake and breathlessness. Our new findings were consistent with a previous cohort study in Singapore that reported soy foods may reduce the development of chronic respiratory symptoms, especially productive cough [12]. It has been suggested that flavonoids from soy foods act as an anti-inflammatory agent in the lung, and can protect against tobacco carcinogens for smokers [12]. However, further research is needed to understand the underlying biological mechanism.

Several limitations should be considered in conjunction with the findings. Habitual dietary assessment was based on self-report using a validated and reliable questionnaire. Nevertheless, responses from the older adults inevitably incurred some recall error due to possible memory and cognitive loss. Therefore, face-to-face interviews were conducted in the presence of patients' next-of-kin to increase the response rate and to improve the accuracy of their answers. In addition, all interviews were conducted by the same investigator (first author) to eliminate inter-interviewer bias. Although the control subjects were recruited during the same period and from the same domain as the prevalent cases and should be representative of the elderly population from which the cases arose, the existence of selection bias could not be ruled out because of their voluntary participation in the study. All participants were blinded to the study hypothesis while the protective effects of soy foods were not yet established for COPD. Reverse causation should not pose a serious threat to the study validity as demonstrated by our sensitivity analysis of the subgroup of patients with COPD diagnosed within one year. Nevertheless, there was still the possibility of residual confounding even though established confounding factors were controlled for in the multivariate analyses.

Another limitation was the exclusion of miso soup and soy sauce towards the calculation of total soy intake. The concentration of miso soup varies substantially according to personal taste, whereas soy sauce is mainly added during cooking and thus difficult to quantify the exact amount consumed. Data were also lacking on less common soy foods such as yuba (soy milk skin) and okara (bean curd byproduct) from the food frequency questionnaire. Finally, evidence concerning the beneficial effects of soy products may be regarded as preliminary, in view of the relatively small number of female participants recruited into the study, even though the gender ratio was typical of the elderly COPD population in Japan.

## Conclusion

Smoking is acknowledged as the major cause of COPD, but the present case-control study has suggested an inverse association between soy products and COPD risk for Japanese adults. More research and/or replications are required to ascertain whether the observed findings can be generalized to other populations, before incorporating these foods into dietary guidelines so as to encourage consumption. Besides experimental studies, long-term prospective cohort studies collecting detailed dietary exposure information are recommended to provide epidemiological evidence on both morbidity and mortality due to COPD.

## Competing interests

The authors declare that they have no competing interests.

## Authors' contributions

FH collected the data and drafted the manuscript. AHL and YZ analysed the data and revised the manuscript. CWB designed the study and revised the manuscript. TH, YT and KN assisted with data collection and interpretation of the findings. HT coordinated the project and helped to draft the manuscript. All authors read and approved the final version of the paper.

## Supplementary Material

Additional file 1 **Lung function results and sensitivity analysis**. Plot of FEV_1 _% predicted against total soy intake, soy consumption and lung function, and soy consumption and COPD risk for the 157 cases diagnosed within 1 year. Click here for file
