# Supplementary material for: Soy consumption and risk of COPD and respiratory symptoms: a case-control study in Japan
Source: Respir Res. 2009 Jun 26;10(1):56. doi: 10.1186/1465-9921-10-56 (PMC2708141; doi:10.1186/1465-9921-10-56)
Supplement: Additional file 1 — Lung function results and sensitivity analysis. Plot of FEV1 % predicted against total soy intake, soy consumption and lung function, and soy consumption and COPD risk for the 157 cases diagnosed within 1 year. [file 1465-9921-10-56-S1.doc]

**Figure S1 - Plot of FEV_1_ % predicted against total soy intake**

**Table S1 - Soy consumption and lung function**

| **Lung function** | **Crude regression coefficient** | **95% CI** | *p* value | **Adjusted regression coefficient*** | **95% CI** | *p* value |
| --- | --- | --- | --- | --- | --- | --- |
| FEV_1_ | 0.004 | (0.002, 0.005) | < 0.001 | 0.002 | (0.000, 0.004) | 0.010 |
| FVC | 0.003 | (0.001, 0.004) | 0.007 | 0.002 | (0.000, 0.004) | 0.014 |
| FEV_1_/FVC | 0.071 | (0.031, 0.110) | < 0.001 | 0.030 | (-0.006, 0.065) | 0.099 |
| FEV_1_ % predicted | 0.124 | (0.068, 0.181) | < 0.001 | 0.069 | (0.016, 0.122) | 0.011 |

* Adjusted coefficients of total soy consumption from linear regression models including age, gender, BMI (5 years ago), education level (high school or below; college or university), alcohol drinking (non drinker; drinker), cigarette smoking (never and ex-smoker; current smoker), smoking pack-years, life-long physical activity involvement (never to not any more involved; always been involved), daily intake of red meat, chicken, fish, vegetables and fruits.

**Table S2 - Soy consumption and COPD risk for the 157 cases diagnosed within 1 year**

| **Variable** | **Cases**  n (%) | **Controls**  n (%) | **OR** * | **95% CI** | *p* value |
| --- | --- | --- | --- | --- | --- |
| **Total soy foods (g/day)** |  |  |  |  | *p* overall = 0.077  *p* trend = 0.003 |
| ≤ 30.43 | 52 (33.1) | 85 (25.1) | 1 |  |  |
| 30.44 - 50.42 | 53 (33.8) | 85 (25.1) | 0.832 | (0.428, 1.617) |  |
| 50.43 - 75.82 | 30 (19.1) | 85 (25.1) | 0.532 | (0.258, 1.094) |  |
| ≥ 75.83 | 22 (14.0) | 84 (24.8) | 0.383 | (0.170, 0.866) |  |
| **Tofu**  **(g/day)** |  |  |  |  | *p* overall = 0.011  *p* trend = 0.001 |
| ≤ 7.53 | 46 (29.3) | 89 (26.2) | 1 |  |  |
| 7.54 - 18.05 | 62 (39.5) | 87 (25.6) | 1.279 | (0.666, 2.459) |  |
| 18.06 - 28.68 | 30 (19.1) | 79 (23.2) | 0.943 | (0.448, 1.987) |  |
| ≥ 28.69 | 19 (12.1) | 85 (25.0) | 0.339 | (0.147, 0.783) |  |
| **Bean sprouts (g/day)** |  |  |  |  | *p* overall = 0.005  *p* trend = 0.051 |
| ≤ 1.67 | 50 (31.8) | 70 (20.6) | 1 |  |  |
| 1.68 - 5.34 | 48 (30.6) | 90 (26.5) | 0.512 | (0.252, 1.038) |  |
| 5.35 - 5.36 | 32 (20.4) | 107 (31.5) | 0.272 | (0.130, 0.568) |  |
| ≥ 5.37 | 27 (17.2) | 73 (21.5) | 0.347 | (0.150, 0.801) |  |
| **Natto**  **(g/day)** |  |  |  |  | *p* overall = 0.381  *p* trend = 0.352 |
| Never | 62 (39.5) | 88 (25.9) | 1 |  |  |
| ≤ 10.70 | 59 (37.6) | 131 (38.5) | 0.677 | (0.367, 1.249) |  |
| 10.71 - 39.30 | 15 (9.6) | 68 (20.0) | 0.502 | (0.220, 1.147) |  |
| ≥ 39.31 | 21 (13.4) | 53 (15.6) | 0.728 | (0.329, 1.613) |  |
| **Soy milk** |  |  |  |  | *p* overall = 0.361  *p* trend = 0.860 |
| Never | 147 (93.6) | 300 (88.2) | 1 |  |  |
| weekly drinkers | 6 (3.8) | 20 (6.0) | 0.427 | (0.130, 1.408) |  |
| daily drinkers | 4 (2.5) | 15 (4.5) | 1.217 | (0.278, 5.323) |  |

* Adjusted odds ratios from logistic regression models including age, gender, BMI (5 years ago), education level (high school or below; college or university), alcohol drinking (non drinker; drinker), cigarette smoking (never smoker; ex-smoker; current smoker), smoking pack-years, life-long physical activity involvement (never to not any more involved; always been involved), daily intake of red meat, chicken, fish, vegetables and fruits.
